# Preoperative Expression Profiles of miR-146a and miR-221 as Potential Biomarkers for Differentiating Benign from Malignant Thyroid Nodules

**DOI:** 10.3390/ijms26157564

**Published:** 2025-08-05

**Authors:** Mervat Matei, Sergiu-Ciprian Matei, Cristina Stefania Dumitru, Roxana Popescu, Ligia Petrica, Ioana Golu, Marioara Cornianu, Isabella Ionela Stoian, Mihaela Maria Vlad

**Affiliations:** 1 Department of Doctoral Studies, “Victor Babes” University of Medicine and Pharmacy, Eftimie Murgu Sq. No. 2, 300041 Timisoara, Romania; mervat.hassan@umft.ro; 2 Abdominal Surgery and Phlebology Research Center, “Victor Babeș” University of Medicine and Pharmacy, 300041 Timisoara, Romania; matei.sergiu@umft.ro; 3 1st Surgical Department, Pius Brînzeu Emergency County Hospital, 300723 Timisoara, Romania; 4 Department II of Microscopic Morphology, Discipline of Histology, “Victor Babes” University of Medicine and Pharmacy, E. Murgu Square, No. 2, 300041 Timisoara, Romania; 5 Department of Cell and Molecular Biology, “Victor Babes” University of Medicine and Pharmacy, 300041 Timisoara, Romania; popescu.roxana@umft.ro; 6 ANAPATMOL Research Center, “Victor Babes” University of Medicine and Pharmacy, Tudor Vladimirescu Street, No. 14, 300174 Timisoara, Romania; 7 Department of Internal Medicine II—Nephrology University Clinic, “Victor Babes” University of Medicine and Pharmacy, Eftimie Murgu Sq. No. 2, 300041 Timisoara, Romania; petrica.ligia@umft.ro; 8 Centre for Molecular Research in Nephrology and Vascular Disease, Faculty of Medicine, “Victor Babes” University of Medicine and Pharmacy, Eftimie Murgu Sq. No. 2, 300041 Timisoara, Romania; golu.ioana@umft.ro (I.G.); vlad.mihaela@umft.ro (M.M.V.); 9 2nd Department of Internal Medicine—Endocrinology Clinic, “Victor Babes” University of Medicine and Pharmacy, P-Ta Eftimie Murgu 2, 300041 Timisoara, Romania; 10 Department of Endocrinology, County Emergency Hospital Timisoara, Blvd. Liviu Rebreanu 156, 300723 Timisoara, Romania; 11 Department of Microscopic Morphology-Morphopatology, ANAPATMOL Research Center, “Victor Babes” University of Medicine and Pharmacy, 300041 Timisoara, Romania; cornianu.marioara@umft.ro; 12 Department of Microscopic Morphology, “Victor Babeş” University of Medicine and Pharmacy, 300041 Timisoara, Romania; isabella.stoian@umft.ro

**Keywords:** thyroid cancer, microRNA, miR-221, miR-146a, circulating biomarkers, molecular diagnostics, preoperative evaluation

## Abstract

Thyroid cancer is the most common endocrine malignancy, and preoperative distinction between benign and malignant nodules remains challenging, especially in cytologically indeterminate cases. Circulating microRNAs (miRNAs) have gained interest as non-invasive biomarkers due to their stability and involvement in tumorigenesis. This study aimed to assess the preoperative diagnostic value of circulating miR-146a and miR-221 in patients undergoing thyroidectomy. A total of 56 patients were included, of whom 24 had malignant and 32 had benign thyroid lesions confirmed by histopathology. Preoperative plasma levels of miR-146a and miR-221 were quantified using qRT-PCR, and relative expression was calculated with the 2^−ΔΔCt^ method. miR-221 expression was significantly higher in malignant cases, with an area under the ROC curve of 1.00, achieving 100% sensitivity and specificity at the optimal threshold. miR-146a showed no significant discriminatory ability. Weak correlations were observed between miRNA expression and clinical parameters such as age, TIRADS score, or thyroid volume. Logistic regression including miR-221 led to perfect separation, indicating strong predictive capacity but precluding multivariate modeling. These findings suggest that circulating miR-221 may serve as a highly accurate biomarker for thyroid malignancy and warrant further validation in larger, prospective cohorts.

## 1. Introduction

Thyroid cancer represents the most prevalent endocrine malignancy worldwide, with an increasing incidence over the last decades largely attributed to improved imaging techniques and widespread use of fine-needle aspiration biopsy (FNAB) [1]. Among its subtypes, papillary thyroid carcinoma (PTC) accounts for approximately 85% of cases and typically carries a favorable prognosis [2]. However, preoperative discrimination between benign and malignant thyroid nodules remains a significant clinical challenge, particularly in cytologically indeterminate lesions (Bethesda categories III and IV), where diagnostic uncertainty may lead to overtreatment or delayed intervention [3].

In recent years, attention has shifted toward molecular biomarkers that may enhance the diagnostic accuracy of conventional methods and offer additional stratification tools. Among these, microRNAs (miRNAs) have emerged as promising candidates due to their stability, detectability in biological fluids, and regulatory functions in tumorigenesis [4].

MicroRNAs are small, endogenous, non-coding RNAs of approximately 19–25 nucleotides that play a critical role in post-transcriptional gene regulation by binding to complementary sequences within the 3′ untranslated regions of target mRNAs [5]. Through this mechanism, miRNAs are involved in a wide range of physiological processes such as cell differentiation, proliferation, apoptosis, and immune response [5,6]. In the context of cancer biology, the dysregulation of specific miRNAs contributes to oncogenic transformation and tumor progression [7]. Among the most studied miRNAs, miR-146a and miR-221 have attracted significant attention due to their dual roles as oncogenes or tumor suppressors depending on the cellular context. Their involvement in modulating inflammatory signaling, epithelial–mesenchymal transition, and cell cycle regulation underscores their potential as valuable diagnostic, prognostic, and therapeutic biomarkers in solid tumors [8,9].

Recent studies have highlighted the diagnostic and prognostic potential of microRNAs (miRNAs)—small non-coding RNAs that regulate gene expression post-transcriptionally—in various human cancers [10,11], including thyroid carcinoma [12]. Among them, miR-146a and miR-221 have emerged as pivotal regulators of tumor proliferation, invasion, and immune modulation, with dysregulated expression reported in PTC tissues and circulating samples [8]. The overexpression of miR-221 has been associated with aggressive behavior and lymph node metastases, while miR-146a may exert context-dependent effects by either promoting or suppressing tumorigenesis [13].

Despite growing evidence supporting the role of these miRNAs in thyroid carcinogenesis, their application as preoperative biomarkers in routine clinical practice remains limited. The majority of available data is derived from post-surgical tissue analyses or cell line studies, with fewer reports evaluating miRNA expression in preoperative FNAB material or peripheral samples obtained prior to thyroidectomy [14]. Moreover, their correlation with final histopathological diagnosis (benign vs. malignant) has not been sufficiently validated in prospective, clinically relevant cohorts.

We selected miR-146a and miR-221 based on their well-documented yet context-dependent roles in thyroid tumorigenesis, and their reported dysregulation in both tissue and circulating samples from patients with papillary thyroid carcinoma (PTC). Prior studies have predominantly focused on miRNA expression in surgical specimens or cell lines, with limited data evaluating their diagnostic performance in preoperative plasma samples [15,16]. The novelty of our study lies in assessing the real-time circulating expression of these two microRNAs in patients undergoing thyroidectomy, using minimally invasive blood sampling and correlating it with final histopathological outcomes. Moreover, our study design captures the potential clinical utility of miR-221 and miR-146a in the pre-surgical setting, especially in cases with indeterminate cytology, where clinical decisions remain challenging. To our knowledge, this is one of a few studies to provide comparative performance metrics for both miRNAs in this specific preoperative context.

In this context, the present study aims to investigate the preoperative expression profiles of miR-146a and miR-221 in patients undergoing thyroid surgery, and to assess their diagnostic utility in differentiating benign from malignant thyroid nodules based on the final histopathological outcome. Our findings may provide insights into the early molecular events of thyroid tumorigenesis and support the development of minimally invasive biomarkers for personalized risk stratification and surgical decision-making.

## 2. Results

### 2.1. Baseline Characteristics

A total of 56 patients were included in this study, with 24 assigned to the study group (patients with histologically confirmed thyroid carcinoma) and 32 to the control group (patients with benign thyroid pathology). The baseline characteristics of the study population are summarized in Table 1. The mean age was slightly higher in the control group compared to the study group (60.3 ± 11.1 vs. 56.6 ± 14.5 years), although the difference was not statistically significant (*p* = 0.394). Regarding gender distribution, females predominated in both groups, with no significant differences observed (78.9% in the control group vs. 73.0% in the study group; *p* = 0.415).

### 2.2. Expression Levels of miR-146a and miR-221

To assess the differential expression of circulating microRNAs between benign and malignant thyroid lesions, we analyzed the relative expression levels of miR-146a and miR-221 using the ΔΔCt method. The comparative analysis between groups was performed using the Mann–Whitney U test, as the data were not normally distributed (Shapiro–Wilk, *p* < 0.05).

As shown in Table 2, the median ΔΔCt for miR-146a was slightly lower in the malignant group compared to the benign group, but this difference was not statistically significant (*p* = 0.301). Similarly, the fold-change (2^−ΔΔCt^) in miR-146a expression was slightly lower in the malignant group (median 1.06; IQR: 0.70–1.55) than in the benign group (median 1.13; IQR: 0.88–1.80), with no significant difference between groups.

For miR-221, both the ΔΔCt values and fold-change showed statistically significant differences between groups (*p* < 0.001). Specifically, patients with malignant lesions had a lower ΔΔCt (reflecting higher expression) and a significantly elevated fold-change (median 1.33; IQR: 0.96–1.78) compared to those with benign pathology (median 0.42; IQR: −0.23–0.86).

These findings suggest a potential diagnostic utility for miR-221, and to a lesser extent for miR-146a, in distinguishing malignant from benign thyroid nodules in the preoperative setting.

**Figure 1:**
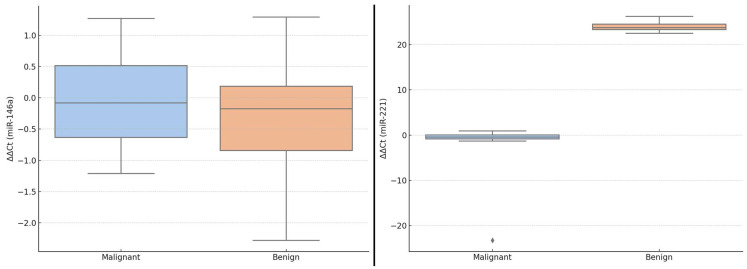
Boxplots showing ΔΔCt values for miR-146a (**left side**) and miR-221 (**right side**) in patients with benign (control group) and malignant (study group) thyroid lesions. Central lines indicate medians; whiskers represent interquartile ranges. The Mann–Whitney U test was used for group comparisons.

These differences in ΔΔCt values are visually illustrated in Figure 1, which displays boxplots of miR-146a and miR-221 expression levels in benign versus malignant thyroid lesions. While the median ΔΔCt of miR-146a was slightly lower in malignant cases, the variability was greater, and the interquartile ranges overlapped. In contrast, miR-221 showed a more pronounced downshift in ΔΔCt values in malignant lesions, indicating higher relative expression. These graphical representations further support the potential diagnostic role of miR-221 in thyroid tumor stratification.

### 2.3. Correlation Between miRNA Expression and Clinical Variables

Spearman correlation analysis was conducted to evaluate the relationship between circulating miRNA expression levels and key clinical or ultrasound parameters, including age, thyroid volume, TIRADS classification, and echogenicity. The results are presented in Table 3. Overall, no statistically significant correlations were identified between ΔΔCt values of miR-146a or miR-221 and the selected clinical variables. A weak positive correlation was observed between miR-146a and the TIRADS score (ρ = 0.19), while miR-221 showed a slight inverse correlation with age (ρ = −0.17) and thyroid volume (ρ = −0.12). Echogenicity showed minimal correlation with either miRNA. Although these associations did not reach statistical significance, the inverse trend between miR-221 and markers of tumor burden (volume, age) may suggest a potential link worth further exploration in larger cohorts.

**Figure 2:**
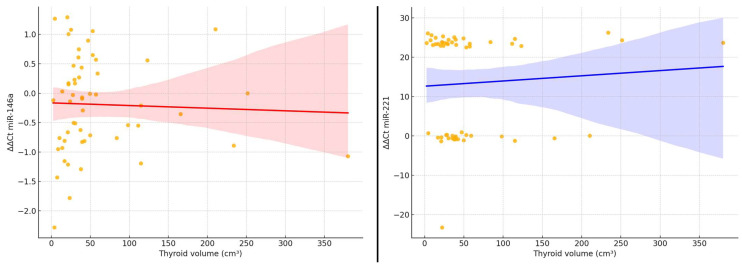
Correlation between estimated thyroid volume (cm^3^) and ΔΔCt expression of miR-146a and miR-221. Linear regression lines are shown in red and blue, respectively. No significant linear correlation was observed (Spearman’s ρ < 0.20, *p* > 0.05).

To further illustrate the relationship between thyroid tumor burden and microRNA expression, we generated scatter plots showing the association between ΔΔCt values of miR-146a and miR-221 and the estimated thyroid volume (Figure 2). A visual inspection of the plots confirms a slight downward trend in ΔΔCt values for miR-221 with increasing thyroid volume, reflecting higher relative expression in larger glands. For miR-146a, the pattern is less consistent, with a broader dispersion of values and no evident trend. Linear regression lines are provided for visual reference; however, neither correlation reached statistical significance (Spearman’s ρ < 0.20, *p* > 0.05).

These findings are consistent with the hypothesis that miR-221 expression may be weakly linked to volumetric disease burden, although the correlation remains modest and should be interpreted with caution.

### 2.4. Diagnostic Performance of miR-146a and miR-221

To evaluate the potential of circulating miRNAs to be diagnostic biomarkers, we assessed the ability of miR-146a and miR-221 to differentiate between benign and malignant thyroid lesions using receiver operating characteristic (ROC) analysis. This evaluation was performed on the entire study population (*n* = 56), including both patients with histologically confirmed malignant tumors (*n* = 24) and those with benign thyroid pathology (*n* = 32).

As shown in Table 4, miR-221 demonstrated excellent diagnostic performance, achieving an AUC (area under the curve) of 1.00, with 100% sensitivity and 100% specificity at an optimal cut-off value of ΔΔCt = 0.91. This suggests that, within this cohort, miR-221 was able to perfectly discriminate between malignant and benign cases.

In contrast, miR-146a exhibited limited discriminative ability, with an AUC of 0.42. At the optimal cut-off (ΔΔCt = 1.27), miR-146a achieved maximal sensitivity (100%) but failed to discriminate benign lesions, with a specificity of only 3%.

**Figure 3:**
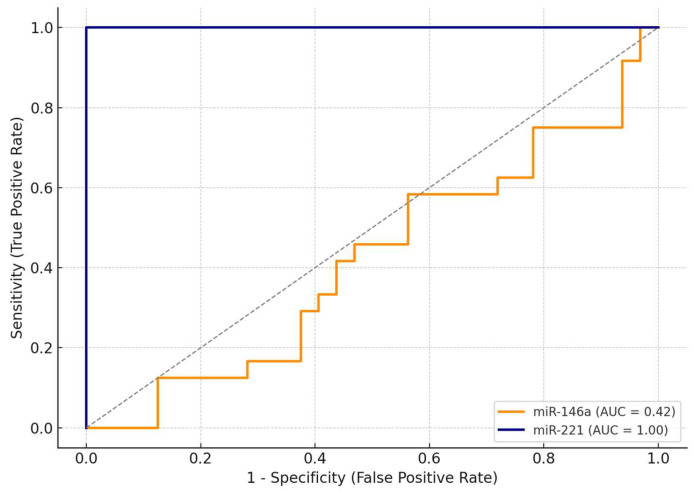
Receiver operating characteristic (ROC) curves comparing the diagnostic performance of miR-146a and miR-221 in differentiating benign from malignant thyroid lesions. The analysis was conducted on the entire study population (*n* = 56), using histopathological diagnosis as the reference standard. miR-221 showed excellent accuracy (AUC = 1.00), while miR-146a had limited discriminative power (AUC = 0.42). The diagonal dotted line represents the line of no discrimination (AUC = 0.5), indicating performance equivalent to random chance.

The comparative ROC curves are illustrated in Figure 3, clearly showing the superior diagnostic accuracy of miR-221. Due to its excellent standalone performance, we did not develop a combined diagnostic model including both miRNAs. These findings highlight miR-221 as a promising non-invasive biomarker for the preoperative identification of malignant thyroid nodules.

Given the perfect classification achieved by miR-221 alone (AUC = 1.00), any attempt to include this marker in a multivariate logistic regression model resulted in perfect separation, rendering the estimation of odds ratios statistically invalid. This outcome confirms the strong predictive capacity of miR-221 in this cohort but simultaneously limits further modeling efforts involving this variable.

To explore the potential contribution of additional clinical parameters, a reduced model excluding miR-221 and including only miR-146a and the TIRADS score was tested. However, this alternative model failed to identify any independent predictors of malignancy (*p* > 0.05), likely due to the limited sample size and the dominant discriminative power of miR-221. Consequently, more extensive cohorts will be necessary to assess the added value of complementary biomarkers and clinical features beyond miR-221.

## 3. Discussion

In this study, we evaluated the preoperative circulating expression of two microRNAs—miR-146a and miR-221—in patients undergoing thyroid surgery for nodular disease. The aim of this study was to assess their potential role as non-invasive biomarkers for distinguishing malignant from benign thyroid lesions.

Among the two microRNAs investigated, miR-221 showed exceptional diagnostic performance, with an AUC of 1.00, 100% sensitivity, and 100% specificity in ROC analysis. This finding aligns with previous studies that highlight miR-221 as one of the most consistently upregulated miRNAs in papillary thyroid carcinoma (PTC), often associated with aggressive tumor behavior and lymph node metastasis [17,18]. The robust separation observed in our cohort confirmed its value as a discriminative marker, potentially complementing or even surpassing current preoperative tools such as TIRADS or fine-needle aspiration biopsy (FNAB) in indeterminate cases.

While the AUC of 1.00 achieved by miR-221 is statistically accurate based on our ROC analysis, we fully acknowledge that such a perfect classification is rare and may reflect overfitting due to the small sample size. All calculations were independently verified, and the diagnostic threshold was determined using the Youden index. Nonetheless, this finding should be interpreted cautiously. Perfect sensitivity and specificity within a limited cohort do not guarantee reproducibility in external populations. The absence of false positives or false negatives in our dataset suggests a strong signal, but larger, prospective studies are essential to confirm this performance and to evaluate its stability across broader clinical scenarios.

Moreover, miR-221 has been functionally implicated in key oncogenic processes in PTC. Experimental studies demonstrated that miR-221 enhances the proliferation, migration, and invasion of PTC cell lines by modulating epithelial–mesenchymal transition (EMT) pathways and targeting the RECK gene (reversion-inducing cysteine-rich protein with Kazal motifs)—a known metastasis suppressor involved in extracellular matrix remodeling [19,20]. Additionally, miR-221 directly downregulates critical cell-cycle inhibitors such as CDKN1B (p27^Kip1^) and CDKN1C (p57^Kip2^), contributing to unchecked cellular proliferation across several cancer types [21]. Furthermore, upregulation of miR-221/222 clusters has been consistently documented in PTCs and correlated with aggressive features, including larger tumor size and lymph node metastases [22]. Together, these findings elucidate the biological basis for our clinical observation and further reinforce the potential of circulating miR-221 as a dual diagnostic and prognostic biomarker in thyroid neoplasia.

Conversely, miR-146a did not show statistically significant differences between benign and malignant groups. Although it exhibited a slightly lower median expression in malignant lesions compared to benign cases, this difference was not statistically significant. This finding is consistent with previous literature that reports the context-dependent roles of miR-146a [23], including both tumor-suppressive and oncogenic effects depending on the cellular microenvironment.

Indeed, miR-146a has been described as a tumor suppressor in various models, where its deletion in mice led to spontaneous sarcomas and lymphomas, suggesting a protective role in tumor initiation [24,25]. Conversely, in thyroid and cervical cancers, miR-146a was found to act as an oncomiR, promoting proliferation and highlighting its dualistic functionality [26]. A comprehensive review noted that miR-146a can serve either as an oncogene or as a tumor suppressor depending on cancer type and cellular context, emphasizing the need for caution when interpreting its expression [27,28].

Our correlation analyses found weak but suggestive associations between miR-221 expression and clinical parameters such as thyroid volume and patient age, implying a potential link with tumor burden. However, these findings did not reach statistical significance. Subgroup analyses based on sex, surgical approach, nodule size, and the TIRADS score failed to reveal consistent expression patterns of either miRNA, although slight differences were observed and may become meaningful in larger cohorts.

A major limitation of this study is the relatively small sample size, which, while sufficient for identifying strong signals such as the diagnostic power of miR-221, may have limited the detection of more subtle associations and precluded the validation of multivariate prediction models. Furthermore, the perfect diagnostic performance of miR-221 (AUC = 1.00) observed in our cohort may reflect an overfitting effect inherent to small, homogeneous populations. Although our results were internally validated through ROC analysis, such an exceptional classification must be interpreted with caution, as it may not be replicated in larger or more heterogeneous cohorts. Future studies should include independent validation sets to assess the external reproducibility and diagnostic robustness of miR-221 in preoperative thyroid nodule evaluation. Attempts to construct a combined logistic regression model including miR-221 resulted in perfect separation, which is a statistical indication of its remarkable discriminative ability but one that inherently prevents the computation of reliable multivariable estimates.

Nevertheless, we acknowledge that the cohort of 56 patients (24 malignant and 32 benign cases) is limited in size and may not capture the full biological and clinical heterogeneity of thyroid nodular disease. Such a relatively small population inherently increases the risk of statistical overfitting and reduces the generalizability of findings across broader or more diverse cohorts. While the current analysis provides preliminary evidence of the diagnostic potential of circulating miR-221, especially in cytologically indeterminate nodules, the robustness of these findings must be interpreted with caution. Further multicenter studies, including larger and more demographically diverse patient populations, are essential to validate and refine these preliminary results before clinical implementation.

In contrast to miR-221, miR-146a demonstrated poor diagnostic performance in our cohort, with an AUC of 0.42 and a specificity of only 3%, despite achieving 100% sensitivity. Such a high false positive rate significantly undermines its clinical utility as a standalone biomarker, as it may lead to unnecessary anxiety and overtreatment in patients with benign nodules. These findings are in line with previous reports that highlight the context-dependent expression and limited reproducibility of miR-146a in circulating samples. Future research should investigate whether miR-146a holds predictive value in combination with other markers or clinical parameters, rather than as an isolated indicator.

Another limitation of our study lies in the lack of significant correlations between circulating miRNA levels and key clinical or ultrasonographic parameters, including patient age, thyroid volume, and TIRADS score. While miR-221 and miR-146a showed weak trends with some variables, none of these reached statistical significance. This relative isolation of the biomarkers from the broader clinical context may hinder their integration into multimodal diagnostic models, particularly those that rely on composite risk stratification tools. These findings underscore the need for larger datasets to determine whether such correlations emerge in more diverse populations.

A further major limitation of this study is the lack of external validation. All data were collected from a single academic center, and the findings were not replicated in an independent cohort. While the diagnostic performance of miR-221 appears promising, its generalizability across different populations, clinical settings, and technical platforms remains uncertain. To confirm its clinical applicability, future studies should involve multicenter collaborations with larger and demographically diverse cohorts, as well as standardized protocols for sample processing and miRNA quantification. External validation will be essential to establish the robustness and reproducibility of miR-221 as a non-invasive preoperative biomarker for thyroid cancer.

Future research should aim to validate these findings in larger, prospectively collected cohorts, which would allow for a more nuanced assessment of both individual and combined biomarker performance. Additional efforts are needed to investigate miRNA panels that encompass both oncogenic and tumor-suppressive profiles, potentially enhancing diagnostic sensitivity and specificity. Moreover, the integration of circulating miRNA expression with ultrasound-based classification systems and cytological findings may significantly improve preoperative risk stratification, particularly in nodules categorized as indeterminate (Bethesda categories III and IV), where clinical decision-making remains most challenging.

## 4. Materials and Methods

### 4.1. Study Design and Patient Selection

This prospective observational study was conducted between 1 July 2024 and 12 December 2024, at the 1st Surgical Department of the “Pius Brînzeu” Emergency County Clinical Hospital, Timisoara. Blood samples were collected prior to surgery, and both molecular and routine clinical analyses were performed within the Clinical Laboratory of Medical Analyses of the same hospital.

The study included adult patients suffering from nodular thyroid disease and scheduled for total thyroidectomy, with available preoperative blood samples and complete histopathological evaluation. The indications for surgery were established by a team made up of endocrinologists and surgeons, and the type of surgery performed was chosen according to existing indications from guidelines and standard protocols [29,30,31,32,33]. In order to maintain uniformity in the study groups, only patients for whom total thyroidectomy was performed were included in this analysis. Exclusion criteria included previous thyroid surgery, ongoing oncologic treatment, thyroid lobectomy, and insufficient sample quality or refusal to participate in the study/sign the informed consent.

Histopathological classification of thyroid lesions post-thyroidectomy served as the reference standard to define the benign or malignant status of each case. All patients provided written informed consent prior to inclusion.

### 4.2. Sample Collection and RNA Extraction

Peripheral blood samples were collected preoperatively in ethylenediaminetetraacetic acid (EDTA)-containing tubes. Total RNA was extracted using the MagMAX™ mirVana™ Total RNA Isolation Kit (Thermo Fisher Scientific, Waltham, MA, USA, Cat. No. A27828), following the manufacturer’s instructions for plasma/serum processing. The procedure was performed in 96-well format, using magnetic bead-based technology to ensure the high yield and purity of small RNAs, including microRNAs. RNA concentration and purity were evaluated using a NanoDrop spectrophotometer (Thermo Fisher Scientific).

### 4.3. Reverse Transcription and Quantitative PCR

Complementary DNA (cDNA) synthesis was performed using the TaqMan™ MicroRNA Reverse Transcription Kit (Applied Biosystems, Waltham, MA, USA, Cat. No. 4366596), with specific stem-loop RT primers for the target microRNAs. Reactions were carried out in a total volume of 15 µL, using 5 ng of total RNA per reaction. The concentration and purity of total RNA were assessed using a NanoDrop™ spectrophotometer prior to cDNA synthesis. Only samples with acceptable 260/280 and 260/230 ratios were included for downstream analysis, ensuring the integrity of small RNAs despite their origin from plasma. The use of 5 ng RNA is consistent with the sensitivity specifications of the TaqMan™ MicroRNA Reverse Transcription Kit, which is optimized for low-input RNA sources, including plasma and serum.

Quantitative PCR (qPCR) was conducted using the TaqMan™ MicroRNA Assays hsa-miR-146a (Assay ID 000468) and hsa-miR-221 (Assay ID 000524), with U6 snRNA (Assay ID 001973) serving as the endogenous control.

Amplification was performed on a QuantStudio™ 5 Real-Time PCR System (Thermo Fisher Scientific), using the TaqMan™ Universal Master Mix II with UNG (Cat. Nos. 4440042 and 4440038) under standard cycling conditions (initial UNG activation at 50 °C for 2 min, polymerase activation at 95 °C for 10 min, followed by 40 cycles of 95 °C for 15 s and 60 °C for 60 s).

### 4.4. Data Processing and Statistical Analysis

Relative quantification of miRNA expression was performed using the comparative Ct method. The expression levels of miR-146a and miR-221 were normalized to the endogenous control U6 small nuclear RNA (snRNA), and ΔCt values were calculated as follows: ΔCt = CtmiRNA − CtU6. For comparisons between benign and malignant cases, ΔΔCt values were computed, and the fold-change in expression was determined using the 2^−ΔΔCt^ method.

All statistical analyses were performed using MedCalc^®^ Statistical Software version 23.1.7 (MedCalc Software Ltd., Ostend, Belgium; https://www.medcalc.org; accessed on 5 July 2025). Continuous variables were tested for normality using the Shapiro–Wilk test. Normally distributed variables were expressed as the mean ± standard deviation (SD) and compared using the unpaired Student’s *t*-test. For non-normally distributed data, the median and interquartile range (IQR) were reported, and comparisons were made using the Mann–Whitney U test.

Categorical variables were expressed as frequencies and percentages and compared using the chi-square test or Fisher’s exact test, as appropriate. Receiver operating characteristic (ROC) curve analysis was used to evaluate the diagnostic performance of miR-146a and miR-221 in discriminating malignant from benign lesions. The area under the curve (AUC), optimal cutoff points (Youden’s index), sensitivity, and specificity were calculated. A two-sided *p*-value < 0.05 was considered statistically significant.

## 5. Conclusions

This study demonstrates that circulating miR-221 has exceptional diagnostic potential for distinguishing malignant from benign thyroid nodules in the preoperative setting. With an AUC of 1.00 and 100% sensitivity and specificity, miR-221 clearly outperformed miR-146a and showed perfect discrimination in ROC analysis. These findings are supported by findings in the previous literature that identify miR-221 as one of the most consistently upregulated microRNAs in papillary thyroid carcinoma, often associated with aggressive histological features and lymph node metastasis. While miR-146a exhibited a trend toward upregulation in malignant cases, it did not reach statistical significance, consistent with its known context-dependent behavior as both an oncogene and tumor suppressor. Correlation analyses revealed weak, non-significant associations between miRNA expression and clinical parameters such as age, thyroid volume, and TIRADS score. Despite the limited sample size, these data suggest that miR-221 may serve as a robust and non-invasive molecular biomarker to complement existing diagnostic tools, especially in indeterminate thyroid nodules. Future studies in larger, prospective cohorts are warranted to confirm these findings and evaluate the integration of miR-221 into multimodal diagnostic algorithms. Incorporating circulating microRNAs into clinical practice may significantly improve early risk stratification and reduce unnecessary surgical interventions.

## Figures and Tables

**Table 1:** Baseline characteristics of the study population.

Variable	Study Group (*n* = 24)	Control Group (*n* = 32)	*p*-Value
Age, years	60.3 ± 11.1	56.6 ± 14.5	0.394
Gender, *n* (%)			0.415
Female	20 (83.3%)	29 (90.6%)	
Male	4 (16.6%)	3 (9.3%)	

**Table 2:** Median values and interquartile ranges (IQRs) of ΔΔCt and 2^−ΔΔCt^ fold-change for miR-146a and miR-221 in malignant versus benign thyroid lesions (study/control group). Data are expressed as the median (25th–75th percentile). Comparisons between groups were performed using the Mann–Whitney U test due to non-normal distribution.

Variable	Study Group (*n* = 24)	Control Group (*n* = 32)	*p*-Value
miR-146a ΔΔCt	−0.08 (−0.63–0.51)	−0.18 (−0.84–0.18)	0.301
miR-146a fold-change (2^−ΔΔCt^)	1.06 (0.70–1.55)	1.13 (0.88–1.80)	0.301
miR-221 ΔΔCt	−0.41 (−0.83–0.05)	23.73 (23.33–24.51)	<0.001
miR-221 fold-change (2^−ΔΔCt^)	1.33 (0.96–1.78)	0.42 (−0.23–0.86)	<0.001

**Table 3:** Spearman correlation coefficients (ρ) between ΔΔCt values of miR-146a and miR-221 and selected clinical and ultrasound parameters. Echogenicity was converted to an ordinal scale (1 = anechoic, 4 = isoechoic). No statistically significant correlations were observed (*p* > 0.05 for all comparisons).

Clinical Parameter	ρ (miR-146a)	ρ (miR-221)
Age (years)	0.14	−0.17
Thyroid volume (cm^3^)	0.12	−0.12
TIRADS score	0.19	−0.00
Echogenicity (1–4 scale)	−0.15	0.03

**Table 4:** Diagnostic performance of circulating miR-146a and miR-221 in differentiating malignant from benign thyroid nodules. The area under the ROC curve (AUC), optimal ΔΔCt cut-off values, sensitivity, and specificity are presented. Malignant cases were coded as “1” and benign as “0” for ROC analysis. Cut-offs were selected using the Youden index.

miRNA	AUC	Optimal Cut-Off (ΔΔCt)	Sensitivity	Specificity
miR-146a	0.42	1.27	1.00	0.03
miR-221	1.00	0.91	1.00	1.00

## Data Availability

The data presented in this study are available on request from the corresponding author.
